# Pediatric Sclerosing Rhabdomyosarcomas: A Review

**DOI:** 10.1155/2014/640195

**Published:** 2014-03-05

**Authors:** Amandeep Kumar, Manmohan Singh, Mehar C. Sharma, Sameer Bakshi, Bhawani S. Sharma

**Affiliations:** ^1^Department of Neurosurgery, All India Institute of Medical Sciences, New Delhi 110029, India; ^2^Department of Neurosurgery, Neurosciences Centre, All India Institute of Medical Sciences, New Delhi 110029, India; ^3^Department of Pathology, All India Institute of Medical Sciences, New Delhi 110029, India; ^4^Department of Medical Oncology, All India Institute of Medical Sciences, New Delhi 110029, India

## Abstract

Sclerosing RMS (SRMS) is a recently described subtype of RMS that has not yet been included in any of the classification systems for RMSs. We did pubmed search using keywords “sclerosing, and rhabdomyosarcomas” and included all pediatric cases (age ≤ 18 years) of SRMSs in this review. We also included our case of an eleven-year-old male child with skull base SRMS and discuss the clinical, histopathological, immunohistochemical, and genetic characteristics of these patients. Till now, only 20 pediatric cases of SRMSs have been described in the literature. Pediatric SRMS more commonly affects males at a mean age of 9 years. Extremeties and head/neck regions were most commonly affected. Follow-up details were available for 16 patients with mean follow-up of 25.3 months. Treatment failure rate was 43.75%. Overall amongst these 16 patients, 10 were alive without disease, 4 were alive with disease, and two died. Thus, overall and disease-free survival amongst these 16 patients were 87.5% and 62.5%, respectively. The literature regarding clinical behaviour and outcome of pediatric patients with SRMSs is patchy. Detailed molecular/genetic analysis and clinicopathological characterization with longer follow-ups of more cases may throw some light on this possibly new subtype of RMS.

## 1. Introduction

Rhabdomyosarcoma (RMS) is the commonest soft tissue tumor of childhood [1] with predilection for head and neck, genitourinary system, and extremities [2]. Though head and neck region is the preferred site for occurrence of RMSs, primary intracranial RMSs are rare tumors with an incidence of around 3% [3]. These are rarely considered in the differential diagnoses of intracranial tumors.

Pediatric RMSs are classified by International Classification for Childhood Sarcomas into embryonal (ERMS), alveolar (ARMS), botryoid, and spindle cell subtypes [4]. Sclerosing RMS (SRMS), a recently described type of RMS, was first reported by Mentzel and Katenkamp in 2000 [5]. Since then 39 cases have been described in the literature, out of which 20 are pediatric cases. We hereby describe a case of right middle cranial fossa SRMS with extracranial extension in an eleven-year-old male child and discuss the literature pertinent to pediatric SRMSs.

## 2. Case Illustration

### 2.1. Clinical Presentation

An eleven-year-old male child presented with complaints of headache for the past 6 months. Patient also had recurrent vomiting of 1 month duration. He also complained of numbness over the right side of face. On examination, patient was conscious and oriented. Sensory loss was present over right V2, V3 distribution of trigeminal nerve. His fundus examination revealed papilloedema. Patient did not have any motor deficits.

### 2.2. Radiology

**Figure 1:**
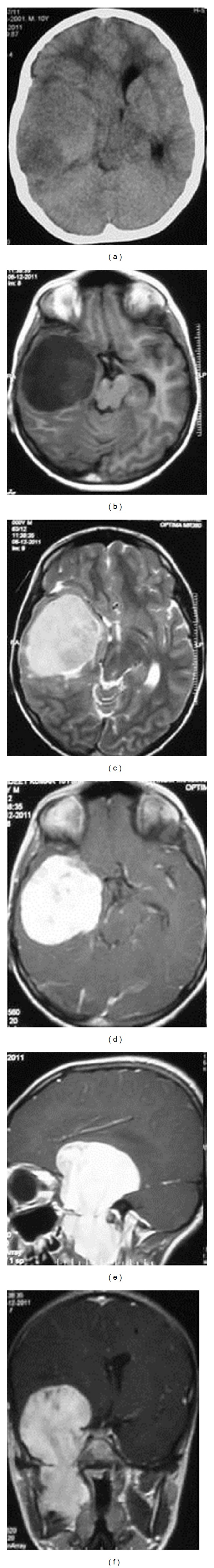
Preoperative images showing a large middle cranial fossa lesion with extension into the infratemporal region (e and f) that is isodense on CT scan (a), hypointense on T1 weighted images (b), hyperintense on T2 weighted images (c), and enhanced homogenously on contrast administration (d, e, and f).

Noncontrast CT scan head revealed an isodense right middle cranial fossa lesion. On MRI scan, the lesion was hypointense on T1 weighted images and hyperintense on T2 weighted images. The lesion was homogenously enhancing. It was extra-axial in location, involved right middle cranial fossa and extended extracranially into the infratemporal fossa. The lesion was creating a significant mass effect with midline shift (Figure 1). Routine haematological investigations and blood chemistry were within normal limits.

### 2.3. Surgery

Patient underwent right frontotemporal craniotomy with zygomatic osteotomy. Intraoperatively, the tumor was present extradurally in right middle cranial fossa which was also extending through the dura into intradural compartment in temporal region as well as the posterior fossa into cerebellopontine angle cistern. The tumor was destroying temporal base and was extending extracranially into subtemporal region. The tumor was highly vascular, firm with good plane of cleavage with brain. The tumor was stuck to the lateral wall of cavernous sinus from which it was separable. The tumor extending into the posterior fossa was excised by drilling the petrous apex. Complete excision of intracranial part of tumor was done. As there was significant bleeding during the surgery, complete excision of infratemporal extension of the tumor was not attempted and only partial decompression was done. The dural defect at middle fossa floor was repaired with temporalis fascia. Postoperative course was uneventful.

### 2.4. Pathological Examination

Tumor tissue in 10% neutral buffered formalin was routinely processed and paraffin embedded. Five microns sections were cut for routine haematoxylin and eosin staining and immunohistochemistry. Immunohistochemical studies were performed using antibodies directed against vimentin (Novocastra, dil, 1 : 100), S-100 (Dako Denmark, dil, 1 : 1500), myogenin (Novocastra, dil 1 : 100), desmin (Dako, dil 1 : 100), and MIC-2 (Novocastra, dil 1 : 200). Labelled streptavidin biotin kit (Universal) was used as a detection system (Dako, Denmark). Antigen retrieval, when required, was performed in a microwave oven. The MIB-1 labelling index (LI) was calculated in the highest proliferating area as the percentage of labelled nuclei per 1000 cell.

### 2.5. Histopathology

**Figure 2:**
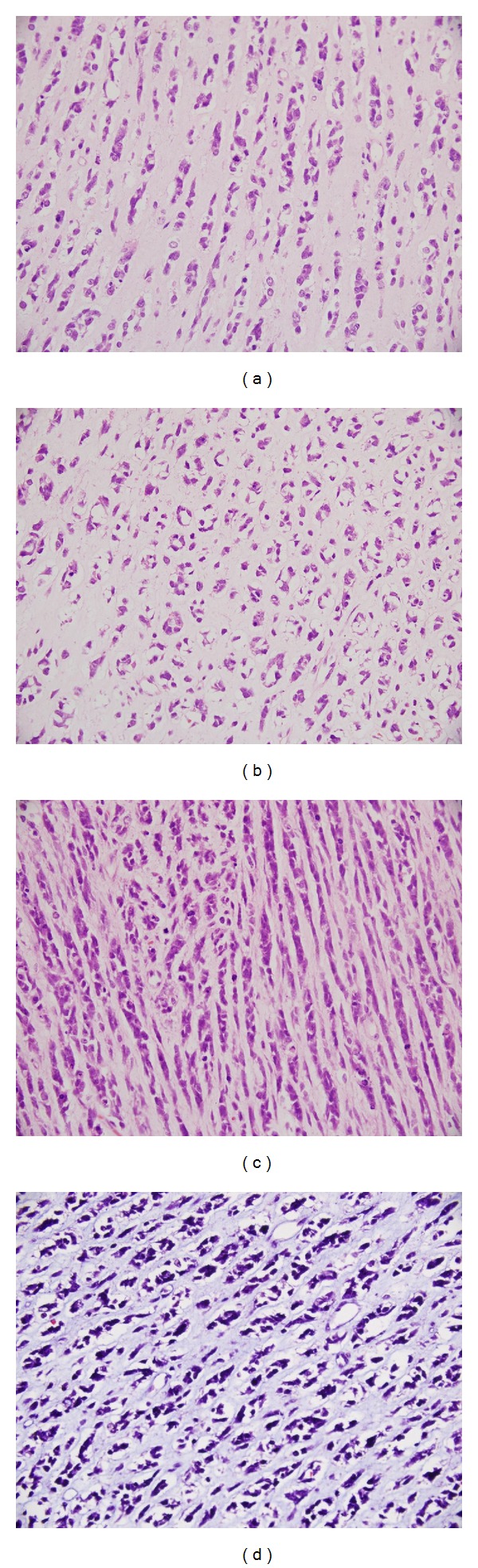
Photomicrographs showing small round cells arranged in Indian file pattern (a), at places showing microalveolar (b) and spindling pattern (c) with hyaline myxoid stroma in the background (H & E ×400). Mitoses are present. The stroma is stained blue with Masson Trichrome stain (d, ×400).

**Figure 3:**
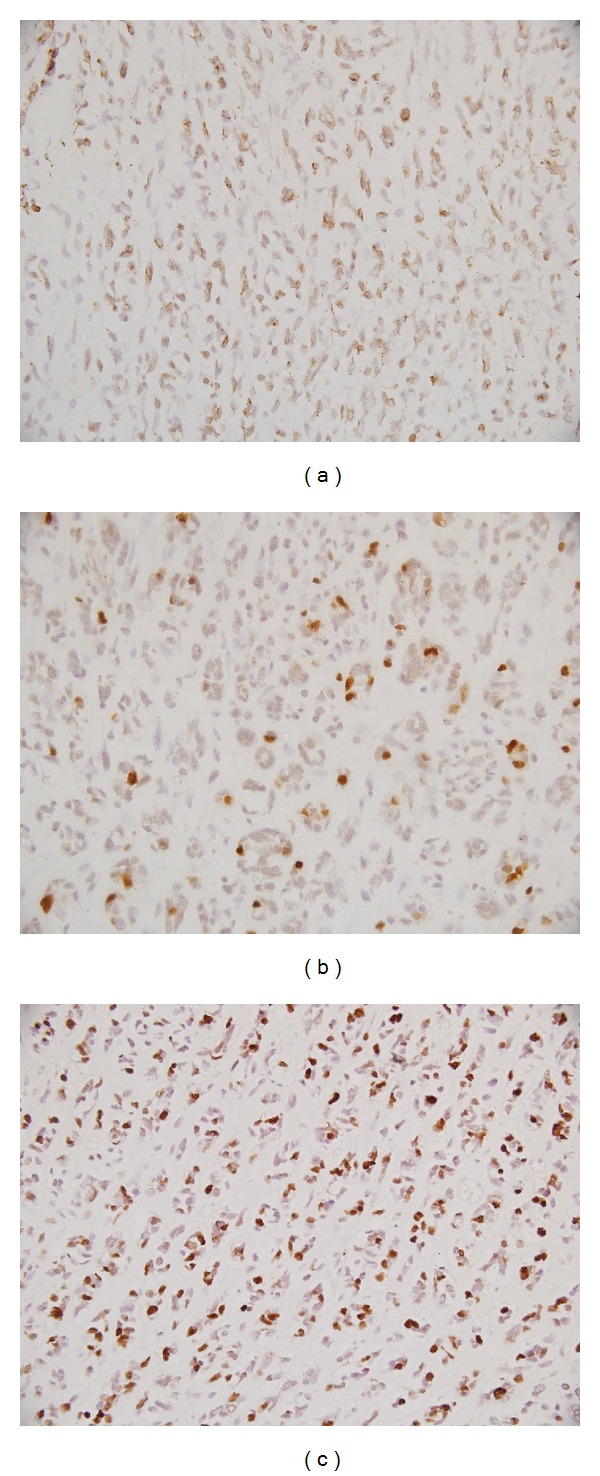
Tumor cells are immunopositive for (a) desmin and (b) focal for myogenin (×400 each). MIB I LI is high (c, ×400).

Microscopic examination showed small round cells arranged in linear fashion (Indian file pattern). At places there were spindling of cells whereas in other areas cells were arranged in microalveolar pattern. The cellular outlines were indistinct with scant amount of cytoplasm. Mitotic activity was brisk. The background stroma was hyalinising and myxoid, at places giving chondroid appearance (Figure 2). No rhabdomyoblasts or strap cells were seen. The tumour cells were immunopositive for desmin (diffuse), vimentin (diffuse), and smooth muscle antigen and focal for myogenin and were immunonegative for s-100, MIC2(CD 99) (Figure 3). MIB 1 LI was 25% in the highest proliferating areas.

Based on above histomorphological features differential diagnoses of alveolar rhabdomyosarcoma and sclerosing rhabdomyosarcoma were kept. The former was excluded in the absence of multinucleated cells with wreath-like arrangement of nuclei, vacuolated or cells with clear cytoplasm and fibrous septae. Moreover myogenin and desmin immunopositivity was focal in comparison to alveolar rhabdomyosarcoma, where it is strong and diffuse. Absence of strap cells, rhabdomyoblasts, and spindle cells and presence of focal microalveolar pattern excluded the possibility of embryonal rhabdomyosarcoma.

Finally based on histomorphological and immunohistochemical features the possibility of sclerosing rhabdomyosarcoma was entertained.

### 2.6. Follow-Up

**Figure 4:**
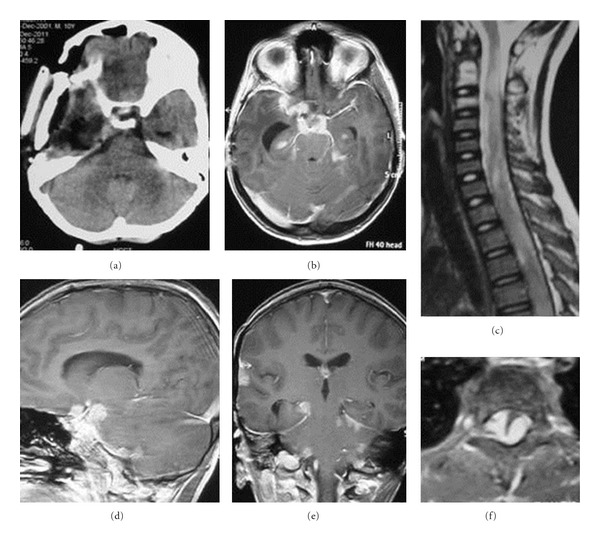
Postoperative images at 1-year follow up showing evidence of previous craniotomy (a). Leptomeningeal metastases are seen both in the intracranial (b, d, and e) and intraspinal (c and f) compartments. The cervical spinal cord is severely compressed by intradural extramedullary metastases (c and f).

Metastatic work-up (including CECT chest and abdomen, bone scan, and CSF cytology) was negative. Patient was given adjuvant radiotherapy and chemotherapy. He received 45 Gy/25# external beam radiotherapy followed by 10 Gy/5# boost to the tumor bed. Patient received 12 courses of vincristine (1.5 mg/m^2^), Actinomycin D (1.35 mg/m^2^), and Cyclophosphamide (2.2 gm/m^2^) (VAC) chemotherapy at 3-week intervals. Postadjuvant therapy CECT head revealed significant reduction in the residual infratemporal tumor. Patient remained asymptomatic till 14 months after initial surgery when he developed rapidly progressive weakness of both lower limbs. Patient was paraplegic and incontinent when he reported again in the clinic. MRI of brain and whole spine revealed disseminated meningeal spread of the tumor. Multiple enhancing intracranial meningeal nodules and metastatic tumor deposits were seen compressing the cervical spinal cord (Figure 4). Patient was subjected to craniospinal radiation (30 Gy/20#). Patient was also given 2 courses of palliative chemotherapy at an interval of 3 weeks (Ifosfamide 2 gm/m^2^ on days 1–3, Carboplatin 500 mg/m^2^ on day 3, Etoposide 100 mg/m^2^ on days 1–3). Further chemotherapy was not continued in view of patient's poor general condition.

## 3. Review of Literature

Since its initial description, 39 cases of SRMS have been described in the literature. Twenty-one of these have been reported in children ≤ 18 years ([6–11] and *present case*) and 17 have been reported in children ≤ 12 years ([6, 7, 9–11] and *present case*). Folpe et al. [8] in 2002 first described a child with this tumor in orbit. Most of these cases have been reported as single case reports except for one large series of 13 cases by Chiles et al. [7].

### 3.1. Clinical Features (Table 1)

#### 3.1.1. Age and Gender

The mean age of children with SRMS described in literature till date was 9 ± 5.17 years (range: 0.3–18 years). Males were affected more frequently than females with a M : F ratio of 1.5 : 1. Our patient was 11-year-old male.

#### 3.1.2. Location

Extremities and head and neck region were involved with equal frequency followed by other sites including retroperitoneum, intra-abdominal, sacral, and scrotal locations. In the present case, the lesion was located in middle cranial fossa with extracranial extension. This is the first case of a skull base SRMS in literature.

#### 3.1.3. Extent of Disease at Presentation

At the time of presentation, all the patients had localized disease and none of the patients for whom clinical details were available had metastases. The size of the lesion varied from 3.2 cm to 10.5 cm.

#### 3.1.4. Treatment of Primary Tumor

Details of treatment given to the patients were available for 7 patients. Five of these patients underwent excision as the initial treatment and 3 of them received adjuvant treatment; two patients received chemotherapy and the present case received both chemotherapy and radiotherapy. Neoadjuvant chemotherapy followed by excision was performed in two patients.

#### 3.1.5. Histopathologic Features (Table 2)

The histopathological features of SRMSs included spindle shaped, polygonal, or round cells embedded in an abundant hyalinising extracellular matrix. Dense acellular hyaline matrix can mimic osteoid or chondroid tissue and sometimes masquerades true identity of the tumor. This has led to its misdiagnosis as chondrosarcoma or osteosarcoma [5, 8]. Another characteristic feature of SRMSs is pseudovascular growth pattern [9–11] for which it can be mistaken as angiosarcoma, chondrosarcoma, or osteosarcoma [8]. Zambrano et al. [11] encountered anaplastic cells with microvascular steatosis resembling lipoblasts. In two of the pediatric cases, rhabdomyoblastic strap cells were seen [7, 11]. The number of mitoses was variable and ranged from 5/10HPF to numerous.

Immunohistochemical staining revealed strong positivity for desmin and MyoD1 in most of the cases, whereas, focal positivity was observed for myogenin and SMA. Variable reactivity was noted for vimentin and CD99. The pancytokeratin, neural, and neuroendocrine markers were negative. The case under discussion showed immunopositivity for vimentin, desmin, and myogenin (focal) and was immunonegative for CD99, SMA, and S-100 protein.

#### 3.1.6. Genetics (Table 2)

There is no specific genetic abnormality attributed to SRMS. Complex karyotype has been observed in 6 patients. Chiles et al. [7] found hyperdiploid karyotype in two of their patients. Croes et al. [12] described the cytogenetic analysis of an adult SRMS with a total of six chromosomal imbalances including gain of chromosomes 1, 11, 16, 18, and 21 and loss of chromosome 22. These changes are observed in ERMS as well [13] and the authors proposed SRMS to be a variant of ERMS. However, Kunhen et al. [14] found three chromosomal imbalances in an adult SRMS, including loss of 10q22 and Y chromosome and trisomy 18. This pattern is unlike that seen in ERMS. Zambrano et al. [11] also reported complex hyperdiploid karyotype in two of their three cases of SRMS. Bouron-Dal Soglio et al. [6] reported for the first time a whole genome analysis of a pediatric SRMS. The analysis revealed a highly complex aneuploid pattern with trisomies of 5, 7, 8, 11, 15, 16, 20, and 22; tetrasomies of 4 and 18, and monosomies of 1, 2, 3, 9, 10, 13, and 14. There was base pair amplification at 12q13-15. This amplification involved HMGA2 and MDM2 genes.

PAX3/PAX7-FKHR gene fusion that is commonly seen in ARMS has been tested in 17 patients with SRMS including both adults and children [6, 7, 9, 11, 12, 14–16]. Only one of these patients has been found to have PAX3-FKHR gene fusion [7].

#### 3.1.7. Follow-Up, Treatment of Recurrences/Metastases, and Outcome (Table 1)

Follow-up details were available for 16 patients. The mean follow-up period was 25.3 ± 21.2 months (range: <1–72 months). Seven patients (7/16; 43.75%) developed local recurrence, distant metastasis, or both during follow-up. Out of these, three patients developed only local recurrence, two patients developed both local recurrence and distant metastases, and two patients developed distant metastasis without local recurrence. The time to recurrence ranged from 3 to 48 months with an average of 19 months. The relation between primary treatment received and treatment failure (local recurrence/metastases) cannot be deduced as the details of the same are lacking in the available literature. Reexcision followed by chemotherapy and reexcision followed by stem cell transplantation were used to treat treatment failures in one patient each, whereas only chemotherapy was used in one of the patients. The present case received craniospinal irradiation and chemotherapy. In the rest three patients with treatment failure, treatment details were not available. Two of the patients with metastases died 12 [8] and 17 months [7] after detection of metastases. One of the patients with local recurrence, treated by reexcision and chemotherapy, was disease-free at short follow-up of 5 months [10]. Rest two patients with treatment failure were alive with disease [6, 7]. Our patient is alive with disease at 16 months.

Overall, amongst the 16 patients for whom information at last follow-up was available, 10 patients were alive without disease, 4 patients were alive with disease, and two patients died (Table 1). Thus, the overall survival amongst these 16 patients, with a mean follow-up of 25.3 months, was 87.5% and disease-free survival was 62.5%.

## 4. Discussion

RMSs are the commonest childhood soft tissue tumors peaking at age < 4years [1]. Different classification systems for RMSs have been described and according to the International Classification for Childhood Sarcomas, pediatric RMSs have been classified into embryonal (ERMS), alveolar (ARMS), botryoid, and spindle cell subtypes [4]. The pleomorphic subtype is predominantly seen in adults [17].

Embryonal and alveolar subtypes were first described in 1894 [18] and 1956 [19], respectively. Stratification into different subtypes is important for prognostication as well as for deciding the management plans as RMSs are heterogenous with respect to clinical, morphological, and molecular characteristics despite sharing the common feature of skeletal muscle differentiation. ERMSs affect infants and young children and involve the genitourinary tract, head and neck, and abdomen. Its histopathology recapitulates all phases of myogenesis, including stellate cells, myoblasts, myotubes, and myofibres [17]. ARMSs have a poorer prognosis and are characterized by alveolar pattern on histopathology with thin fibrous collagenous septae that support the tumor cells and separate the tumor cell nests [17]. They commonly affect extremities and trunk in adolescents. The dense hyaline matrix is not a feature of these variants of RMS. ARMSs show a characteristic translocation involving PAX genes located on chromosomes 1 and 2 and FKHR gene located on chromosome 13 [17]. On the other hand, no specific genetic abnormality has been found in ERMSs; however, an allelic loss of 11p15 and a hyperdiploid pattern have been described [7, 12].

Recently in 2000, Mentzel and Katenkamp [5] described three unusual cases of RMS with unique histopathological features of hyaline sclerosis and pseudovascular pattern. Folpe et al. [8] in 2002 described another four such similar cases. The atypical histopathological features of prominent hyaline osteoid or chondroid matrix, pseudovascular pattern, lack of alveolar pattern, and absence of gene fusion products characteristic of ARMS (Pax3/FKHR and Pax7/FKHR) defied classification into preexistent types of RMS. These tumors also show a strong positivity for Myo D1 and weak positivity for myogenin that is not typical of ARMS. Due to these features, Folpe et al. [8] proposed these tumors to be either a variant of ERMS or a new subtype of RMS and named these tumors as “*sclerosing RMS*”. However, SRMS lacks the typical features of ERMS as well.

Though its existence as a separate entity is still debatable, with some authors reporting similarities with ERMS [8, 12] or ARMS [5, 7, 14], more and more cases are being identified and described in the literature.

SRMS shares clinicopathological features both with ERMS and ARMS. The presence of strap cells [5, 8, 11], involvement of urinary bladder [11], the presence of areas with spindle cells [7, 11], more prominent expression of MyoD1 over myogenin [5, 7–9, 11], and gain of chromosomes on karyotyping in some cases point towards ERMS [7, 11, 12]. However, common extremity involvement is unusual for ERMSs [5, 8].

The features pointing towards ARMS include occurrence in adults, frequent involvement of extremities, and presence of occasional microalveolar pattern and primitive round cells [5, 7, 8, 12]. However, the absence of pseudoalveolar pattern and presence of prominent hyaline matrix in place of thin fibrous septae do not favour the alveolar pathology [7, 8, 12]. The weak myogenin expression is also in contrast to what is typical of ARMS and the characteristic fusion gene *PAX/FXHR *of ARMS has not been seen except in only one of the cases [7].

In our case also, initially there was a diagnostic dilemma but immunohistochemical analysis helped in deciphering the true identity of tumor.

The 10-year survival rates for spindle and botryoid variants of ERMS, ERMS, and ARMS are 80–86%, 55%, and 9%, respectively [20]. The overall survival of small number of pediatric cases of SRMS (16 patients with available follow-up details) with a short follow-up of <2 years is 87.5%. It is very difficult to arrive at any conclusion with such a small number of patients and very short follow-up period; however, longer follow-up studies may reveal true aggressiveness and malignant potential of SRMS. Since the review lacked treatment details, based on what is known about RMS, all such patients should be treated like other types of RMSs with aggressive chemotherapy, radiotherapy, and surgical resection, keeping in mind that treatment failure rate for pediatric SRMS is high (43.75%).

## 5. Conclusion

SRMS is a recently described entity that is still waiting to be given recognition of a new subtype of RMS. Its shared clinicopathological features with ARMS and ERMS and lack of any characteristic genetic marker make definite categorization of SRMS as a new subtype difficult. Also the literature regarding the clinical behaviour and outcome is patchy. Detailed molecular/genetic analysis and clinicopathological characterization with longer follow-ups of more cases may throw some light on this possibly new subtype of RMS.

## Figures and Tables

**Table 1:** Showing clinicopathological features of 21 cases of pediatric sclerosing rhabdomyosarcomas reported in the literature (including present case).

Case number	Author	Year	Age/ sex	Site	Size (cm)	Mets (1)	Primary Rx	Adj. Rx	Local recurrence	Mets (2)	TTR/TTM (mos)	Rx of Rec/Mets	F/U (mos)	Outcome
1	Folpe et al. [8]	2002	18/M	Orbit	NA	(−)	Excision	(−)	(−)	Intra-abdominal and lymph nodes	48	CT	60	Died

2	Vadgama et al. [9]	2004	3/F	Ischiorectal fossa/buttock	3.2	(−)	Excision	CT	(−)	(−)	(−)	(−)	4	AWD

3	Chiles et al. [7]	2004	6/M	Arm	NA	NA	NA	NA	(+)	(−)	20	NA	39	Alive with disease
4			11/M	Elbow	NA	(−)	NA	NA	(−)	(+)	20	NA	37	Dead
5			7/F	Pharynx	NA	(−)	NA	NA	(+)	(−)	4	NA	16	Alive with disease
6			10/F	Orbit	NA	(−)	NA	NA	(−)	(−)	(−)	(−)	18	AWD
7			3/M	Leg	NA	(−)	NA	NA	(−)	(−)	(−)	(−)	10	AWD
8			8/F	Sinus	NA	(−)	NA	NA	(−)	(−)	(−)	(−)	<1	AWD
9			16/F	Thigh	NA	(−)	NA	NA	(−)	(−)	(−)	(−)	25	AWD
10			4/F	Scapula	NA	(−)	NA	NA	(−)	(−)	(−)	(−)	NA	NA
11			12/M	Masseter	NA	(−)	NA	NA	(−)	(−)	(−)	(−)	<1	AWD
12			9/M	Orbit	NA	(−)	NA	NA	(−)	(−)	(−)	(−)	43	AWD
13			4/M	Neck	NA	(−)	NA	NA	(−)	(−)	(−)	(−)	37	AWD
14			0.3/M	Retroperitoneum	NA	(−)	NA	NA	(−)	(−)	(−)	(−)	NA	NA
15			5/M	Scrotum	NA	(−)	NA	NA	(−)	(−)	(−)	(−)	24	AWD

16	Zambrano et al. [11]	2006	8/F	Intra-abdominal	10.5	(−)	CT f/b Excision	(−)	NA	NA	NA	NA	NA	NA
17			18/M	Thigh	NA	NA	NA	NA	NA	NA	NA	NA	NA	NA
18			17/M	Lower leg	9.0	NA	CT f/b Excision	NA	NA	NA	NA	NA	NA	NA

19	Wang et al. [10]	2008	12/F	Nasal cavity	4.0	(−)	Excision	(−)	(+)	(−)	3	Excision + CT	5	AWD

20	Bouron-Dal Soglio et al. [6]	2009	7/M	Deltoid	3.7	(−)	Excision	CT	(+)	Lung, Lymph nodes	24	Excision + SCT	72	Alive with Disease

21	Present case	2013	11/M	Skull base	9.3	(−)	Excision	CT + RT	(+)	Spinal leptomeningeal	14	RT + CT	16	Alive with Disease

AWD: alive without disease; CT: chemotherapy; RT: radiotherapy; NA: not available; Mets (1): metastases at presentation; Mets (2): metastases during follow-up; Adj.: adjuvant; Rx: treatment; f/b: followed by; SCT: stem cell transplantation; mos: months; Rec: recurrence; TTR: time to recurrence; TTM: time to metastases.

**Table 2:** Histopathological, immunohistochemical, and genetic features of 21 cases of pediatric sclerosing rhabdomyosarcomas.

Year	Author	Age/ Sex	Vimentin	Desmin	Myogenin (myf4)	MyoD1	SMA	Cytokeratin	S100	CD99	Amount of fibrosis	Rhabdo-myoblasts	Mitoses	PAX3/FXHR by RT-PCR	Karyotype
2002	Folpe et al. [8]	18/M	NA	Focally (+)	Focally (+)	Strongly (+)	3+	Neg.	Neg.	Neg.	Abundant	(−)	>25/20 HPF	NA	NA

2004	Vadgama et al. [9]	3/F	Strongly (+)	Strongly (+)	Focally (+)	Strongly (+)	Focally (+)	Neg.	Neg.	Focally (+)	Dense	(−)	32/100 HPF	Neg.	NA

2004	Chiles et al. [7]	6/M	NA	Pos.	Pos.	Pos.	NA	NA	NA	NA	>50%	(−)	NA	NA	NA
		11/M	NA	NA	NA	NA	NA	NA	NA	NA	10–50%	(−)	NA	Neg.	** CK**
		7/F	NA	Focally (+)	Focally (+)	Strongly (+)	NA	NA	NA	NA	>50%	(−)	NA	NA	NA
		10/F	NA	Strongly (+)	Focally (+)	Strongly (+)	NA	NA	NA	NA	>50%	(−)	NA	Neg.	NA
		3/M	NA	Strongly (+)	Focally (+)	Strongly (+)	NA	NA	NA	NA	>50%	(−)	NA	Pos.	NA
		8/F	NA	Strongly (+)	Focally (+)	Strongly (+)	NA	NA	NA	NA	>50%	(−)	NA	NA	NA
		16/F	NA	Focally (+)	Focally (+)	Strongly (+)	NA	NA	NA	NA	>50%	(−)	NA	Neg.	NA
		4/F	NA	Strongly (+)	Strongly (+)	Strongly (+)	NA	NA	NA	NA	10–50%	(−)	NA	NA	NA
		12/M	NA	Strongly (+)	Focally (+)	Strongly (+)	NA	NA	NA	NA	>50%	(−)	NA	Neg.	NA
		9/M	NA	Focally (+)	Strongly (+)	Strongly (+)	NA	NA	NA	NA	10–50%	(−)	NA	NA	** CK**
		4/M	NA	Strongly (+)	Strongly (+)	Strongly (+)	NA	NA	NA	NA	10–50%	(−)	NA	NA	NA
		0.3/M	NA	Focally (+)	Strongly (+)	Strongly (+)	NA	NA	NA	NA	10–50%	(−)	NA	NA	NA
		5/M	NA	Strongly (+)	Focally (+)	Focally (+)	NA	NA	NA	NA	>50%	(+)	NA	Neg.	NA

2006	Zambrano et al. [11]	8/F	NA	Strongly (+)	Focal (+)	Focally (+)	Focally (+)	NA	Neg.	Neg.	Abundant	(−)	Numerous	Neg.	** NK**
		18/M	NA	Strongly (+)	Weakly (+)	Strongly (+)	Strongly (+)	NA	Neg.	Weakly (+)	Abundant	(−)	Numerous	Neg.	** CK**
		17/F	NA	Focally (+)	Focally (+)	Focally (+)	Focally (+)	NA	Neg.	Weakly (+)	Abundant	(+)	Numerous	Neg.	**CK**

2008	Wang et al. [10]	12/F	Strongly (+)	Focally (+)	Focally (+)	Strongly (+)	Focally (+)	NA	Pos.	NA	Abundant	(−)	5/10 HPF	NA	NA

2009	Bouron-Dal Soglio et al. [6]	7/M	NA	Strongly (+)	Focally (+)	NA	NA	NA	NA	NA	Abundant	(−)	NA	Neg.	** CK**

2013	Present case	11/M	Strongly (+)	Strongly (+)	Focal (+)	Not done	Neg.	Neg.	Neg.	Neg.	Abundant	(−)	NA	Not done	Not done

NA: not available; Neg.: negative; Pos.: positive; HPF: high power field; CK: complex karyotype; NK: normal karyotype.
